# The Promise of Digital Biopsy for the Prediction of Tumor Molecular Features and Clinical Outcomes Associated With Immunotherapy

**DOI:** 10.3389/fmed.2019.00172

**Published:** 2019-07-31

**Authors:** Giuseppe Luigi Banna, Timothée Olivier, Francesco Rundo, Umberto Malapelle, Filippo Fraggetta, Massimo Libra, Alfredo Addeo

**Affiliations:** ^1^Oncology Department, United Lincolnshire Hospital Trust, Lincoln, United Kingdom; ^2^Oncology Department, University Hospital Geneva, Geneva, Switzerland; ^3^ADG Central R&D - STMicroelectronics of Catania, Catania, Italy; ^4^Department of Public Health, University Federico II of Naples, Naples, Italy; ^5^Department of Pathology, Cannizzaro Hospital, Catania, Italy; ^6^Oncologic, Clinic and General Pathology Section, Department of Biomedical and Biotechnological Sciences, University of Catania, Catania, Italy

**Keywords:** radiomics, pathomics, omics, predictive, immunotherapy, cancer, digital biopsy, prognostic

## Abstract

Immunotherapy by immune checkpoint inhibitors has emerged as an effective treatment for a slight proportion of patients with aggressive tumors. Currently, some molecular determinants, such as the expression of the programmed cell death ligand-1 (PD-L1) or the tumor mutational burden (TMB) have been used in the clinical practice as predictive biomarkers, although they fail in consistency, applicability, or reliability to precisely identify the responding patients mainly because of their spatial intratumoral heterogeneity. Therefore, new biomarkers for early prediction of patient response to immunotherapy, that could integrate several approaches, are eagerly sought. Novel methods of quantitative image analysis (such as radiomics or pathomics) might offer a comprehensive approach providing spatial and temporal information from macroscopic imaging features potentially predictive of underlying molecular drivers, tumor-immune microenvironment, tumor-related prognosis, and clinical outcome (in terms of response or toxicity) following immunotherapy. Preliminary results from radiomics and pathomics analysis have demonstrated their ability to correlate image features with PD-L1 tumor expression, high CD3 cell infiltration or CD8 cell expression, or to produce an image signature concordant with gene expression. Furthermore, the predictive power of radiomics and pathomics can be improved by combining information from other modalities, such as blood values or molecular features, leading to increase the accuracy of these models. Thus, “digital biopsy,” which could be defined by non-invasive and non-consuming digital techniques provided by radiomics and pathomics, may have the potential to allow for personalized approach for cancer patients treated with immunotherapy.

## Introduction

In the *data deluge* era, there is a unique opportunity to explore biological processes at multiple scales. Deriving useful information from data, often poorly structured, at large scales, led to the emergence of the so-called “-*omics*” disciplines (genomic, transcriptomic, proteomic, metabolomic, etc.) (1). Powerful bioinformatic tools allow for high-throughput extraction processes that convert images into data, from which biostatistical analysis, combined with clinical or other “*-omics*” data, may enhance diagnostic accuracy and find new predictive or prognostic factors (2). Applied to radiological images (most often computed tomography [CT], magnetic resonance [MR] imaging, and positron-emission tomography [PET]), it is called radiomics, which has been the pioneer in the field of images data analysis. Pathomics, that is a more recent discipline, ensues when the same processes are being applied to histopathological images.

In this review, we describe the basic background based on which these new disciplines have emerged and the important steps involved in imaging acquisition to clinical supporting correlations. Selected radiomics and pathomics reports will illustrate achievements in this field, with a focus on immunotherapy. Challenges and future development will be then considered.

## Background for Radiomics and Pathomics

The founder hypothesis supporting the use of radiomics and pathomics in medical care is that data derived from images have a correlation with the underlying biological processes. More precisely, data derived from images would give additional information in relation with the underlying biological processes in comparison with the visual interpretation of the image as a picture, which is the traditional way of interpreting images (3).

Radiomics and, at a lesser extent, pathomics, fill the need to assess tumor heterogeneity. The presence, within the tumor, of distinct molecular cell clones, is a hallmark of cancer physiopathology (4). Natural history of cancer, as well as resistance mechanisms acquired through therapeutic selective pressure, manifest spatial and temporal heterogeneity of tumor cells (5, 6). Addressing tumor heterogeneity is one of the major goals of new therapeutic approaches and blood biomarkers may present limitations that could be overcome by radiomics and pathomics. In particular, radiomics represents a promising non-invasive and repeatable tool during the course of the disease.

Furthermore, traditional medical practice, based on human visual interpretation of images, is known to be inaccurate in up to 20% of cases in radiology and almost the same discrepancy rates are found in pathology reports (2). Despite many explanations accounting for these reporting errors, the result is the high prevalence of diagnosis unreliability, with clinical consequences for patients.

As far as cancer immunotherapy is concerned, immune checkpoint inhibitors (CPIs) have emerged as an effective therapeutic option for patients with aggressive tumors such as lung cancer (7, 8), although a few patients seem to benefit from the long-term benefit from this treatment (9). Aiming at identifying these patients, the expression of programmed cell death ligand-1 (PD-L1) has been widely explored as a predictive biomarker with contrasting results across different tumor subtypes and several methodological issues, mostly related to its variability and spatial intratumoral heterogeneity, that have been undermining its role and use (10). Other predictive biomarkers, such as the tumor mutational burden (TMB), are currently poorly applicable in the clinical practice and, noteworthy, identify a different sensitive population from the one selected by the PD-L1 (11). Thus, there is a need for new biomarkers to integrate into clinical practice in order to early identify patient response (or progression) to CPIs and avoid their potential sever toxicity (12–14).

## Process Description and Methods

**Figure 1:**
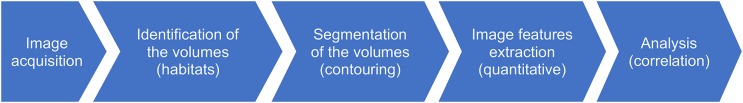
Essential steps of the radiomics/pathomics process.

Every “-omics” analysis requires a multistep process. Each stage has its own specificities. Radiomics process has been established as a model for other disciplines in image data analysis (such as pathomics) and essentially consists in the following five steps: image acquisition, identification of the target volumes, segmentation of the volumes, features extraction from the volumes and analysis [see Figure 1; (3, 15)].

After the first step, the identification of the volumes must identify tumor location and determine distinct parts within the tumor. These regions will be called *habitats*, and present specific biological properties (blood flow, cell density, edema, necrosis). Image data analysis can help to identify such *habitats* (16) before data extraction. This step is intentionally done before data extraction, thus giving additional data that would not be automatically detected by subsequent data analysis (17).

The next step, the most critical one, is the segmentation. It consists in contouring the volumes of interest. Its importance derives from the fact that all the data extraction process will be generated by each segmented volume, and any error at this point could mislead further interpretation. Given inter-operator variability and the time consuming of manual delineation, semi-automated tools seem to be the most reliable and cost-effective approaches to this step (18).

Next stages, highly technical, allow for high-throughput extraction of quantitative data and their analysis. Data extraction results in image-based “features.” These features are mathematically and bioinformatically derived from images through first-, second-, or higher order statistical processes.

Radiomics features could be “texture” feature, “tumor heterogeneity” feature, etc. Quantitative features may be presented based on histograms for each volume of interest.

Analysis of radiomics features, along with clinical data or other “*-omics*” data try to find correlations with biological processes. The analysis aims to define and validate image-derived features as biomarkers that could have prognostic or predictive values helping thus to support medical decisions.

Different methods could apply to exploit this process, but we will exclusively describe, as an example to understand the full operation, the bio-inspired system we have been currently investigating within a multi-disciplinary joint lab (engineers, mathematicians, and clinicians) for pathomics and radiomics. The mathematical core is based on recent Machine Learning (ML) approaches. The high capability of the ML systems in addressing complex problems and, in particular, those related to healthcare and medical applications, has already been confirmed (19, 20). As an additional validation, we have also implemented a joined mathematical-ML system for the early discrimination of skin lesions by dermoscopic images with high diagnostic accuracy (21). The bio-inspired system is based on the correlation between the tumor aggressiveness and fractal dimension of the related lesions (22).

**Figure 2:**
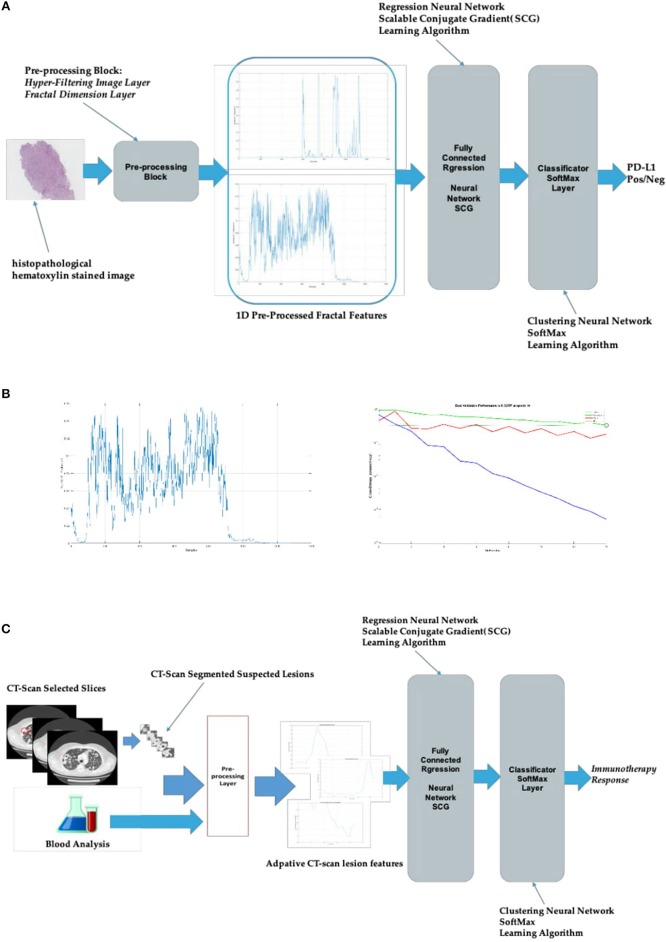
Bio-inspired system for radiomics and pathomics. **(A)** Bio-inspired system for pathomics in lung cancer; **(B)** the diagram on the left shows an example of fractal dimension time–series extracted from a single histopathological hematoxylin stained image. The one on the right illustrates the learning dynamic of the system during the training session: the lower (blue) curve shows the training dynamic (i.e., the progressive error reduction) while the middle (red) and the upper (green) curves show the testing and validation, respectively; **(C)** bio-inspired system for radiomics in urothelial cancer. The pre-processing input data used arise from CT-scan images and blood analysis data.

Currently, we have been testing this approach within two specific subject areas. The first one in the field of pathomics for lung cancer (reported in Figure 2A), regards specifically the prediction of PD-L1 overexpression (a biomarker predictive of response to immunotherapy in this tumor subtype) by the analysis of histopathological hematoxylin stained images; this could represent a useful guide to pathologists (and physicians). The second one concerns radiomics for urothelial cancer and it is aimed to correlate tumor response to immunotherapy with CT-scans medical images and other blood data (i.e., radiomics).

Starting from these premises, for pathomics, we have implemented a hyper-filtering pre-processing of histopathological hematoxylin stained images (Figure 2A). Each of the analyzed images has been converted from RGB (red-green-blue) color spaces to luminance (*Y*) chrominance information (CbCr) spaces with the divided gray-level representation of the histopathologic image. The luminance *Y* gray-level images have been then pre-processed by the hyper-filtering layer inside the “Pre-processing Block” using an *ad-hoc* adaptive thresholds-based approach in order to obtain a 1D representation of the source gray-level *Y* images. From every pre-processed *Y* images, the system computes the corresponding fractal dimension according to the Hausdorf model allowing to obtain, through an additional computing analysis, a time-series collection of those fractal dimensions (23). These pathomics features, ensued along with histopathologic image-features extracted by the AutoEncoder system (that is designed with one hidden layer of 20 neurons) also included in the “Pre-processing Block” are fed into a regression neural network learned by a classical Scalable Conjugate Gradient (SCG) back-propagation algorithm, with the final classification layer based on the SoftMax approach (21).

For the learning process (training phase), the authors used 70 percent of the histopathologic images while the remaining 30 percent serves for testing and validation. The learning dynamic of the bio-inspired system and an example of the fractal dimension time-series extracted from images are represented in Figure 2B.

For our radiomics project, the system is basically the same as above described (Figure 2A) with the input being the sequence of segmented CT-scan slices in which the lesion is visible along with the possible association of normalized representation of laboratory data (i.e., blood values). Through an innovative patented approach, time-series mapped signals are extracted in the pre-processing layer, starting from an *ad-hoc* analysis of the morpho-geometric dynamic of the CT-scan lesion in each of the slices. The resulting output (time-series data) feed, as a new input, the regression neural layer and then the SoftMax classificatory, which finally provide the binary discrimination of the positive or negative response to the immunotherapy (Figure 2C).

## Radiomics and Pathomics Applications

### Diagnosis (Early) and Classification

Computer-aided diagnosis and detection system (CAD) help for better detection and diagnostic accuracy (24). Radiomics analysis, although sharing some principles with CAD, do not answer only a precise question (detection) but it is a complex process looking for a correlation with biological mechanisms. Magnetic resonance (MR) images from 147 patients with confirmed prostate cancer showed that several MR derived “texture” features were significantly different in benign and malignant prostate tissue and in samples with different Gleason scores (25). Another study confirmed that texture features extracted from MR prostatic images could define with accuracy not only the Gleason score but also score patterns: two patterns of Gleason score 7 (“4 + 3” vs. “3 + 4”) were correctly discriminated with 92% accuracy (26).

Pathomics studies were preceded by computer-aided-system tools, with for instance a fractal analysis set, showing powerful discrimination in grading prostatic cancer (27). In another study, analysis from 39 patients with colorectal lesions finds that analysis of multiscale texture features, extracted through a “3D wavelet transform filter” from histopathological images, were able to correctly distinguish different colorectal cancer grades (28).

In the context of immunotherapy, Tang et al. associated radiomics features with PDL1 expression and CD3 count in two cohorts (training and validation cohort of *n* = 114 and *n* = 176, respectively) lung cancer patients (29). Sun et al. developed a radiomic signature for tumor-infiltrating CD8 cells in a retrospective multicohort study on overall 491 patients with advanced solid tumors (30).

### Prognosis

The prognostic value of radiomics was reported in 108 patients with lung adenocarcinoma (separated in two independent cohorts), radiomics features (including tumor shape complexity and intratumor density variation) were strongly correlated with overall survival (31). Furthermore, Aerts et al. analyzed 440 image-related features extracted from CT images of 1019 patients with lung or head and neck cancer. They could find many radiomic features having a prognosis value and built a prognostic radiomic signature, which was found to be correlated with underlying gene-expression patterns (32).

Pathomics could also yield prognostic information. Pathomics features derived from the analysis of 2186 histopathological images were explored to distinguish short-term and long-term survivors in patients with non-small lung cancer. The survival prediction model was validated on 294 additional images (33).

Pathomics and radiomics studies in glioblastoma patients illustrated how correlations derived from different data scales (neuroimaging, pathologic and genomic) may give a deeper understanding of tumor biology and predict clinical outcomes (34–37).

Regarding immunotherapy, in the above-mentioned study of Tang et al. (29), a radiomic immune pathology-informed model was developed. The model defined four subsets of lung cancer patients significantly associated with overall survival. A group of patients with favorable prognosis was identified, harboring low CT intensity and high heterogeneity (as radiomic features) and low PDL1 with high CD3 infiltration, indicating a favorable immune activity.

### Outcome Prediction

To date, fewer works have explored the predictive value of radiomics and pathomics features. MR images-derived texture features from 58 breast cancer patients showed that radiomic features before neoadjuvant chemotherapy could predict response (38).

As far as immunotherapy is concerned, in the study of Sun et al. (30), the radiomic-based biomarker of tumor-infiltrating CD8 cells was validated in 3 independent cohorts and showed predictive value for tumor response to the anti-PD-1 or anti-PD-L1 therapy. Moreover, Colen et al. elaborated a two-feature radiomic model in order to predict immunotherapy-induced pneumonitis characterized by strong internal accuracy (100%) (39).

## Future Challenges of Image-Derived Features

Some challenges regarding the multistep process of radiomics and pathomics still need to be adequately addressed. Methodologically, quantitative image-derived features biomarkers should undergo a multicenter prospective trial to be validated, as it is for other biomarkers. Technically, each step of image data analysis needs proper benchmarking and reproducibility. Furthermore, curation of big data, time processing and data sharing are other major challenges. In this sense, great efforts have been made by the scientific community to share tools (software, web-based platforms) allowing physicians to explore image data analysis (40–42). The Quantitative Imaging Network, for instance, initiated in 2008 and supported by the National Cancer Institute, is an example of the importance of these new disciplines. Along with the identification of biological biomarkers, assessed by longitudinal repeated tumor samples taken by tissue biopsy and/or liquid biopsy, we postulate that “digital biopsy,” as previously defined, could allow to find potential correlation between biological biomarkers and “radiomics and pathomics biomarkers,” and have the potential to better define prognosis and prediction of response. Interdisciplinarity and integration within “-omics” disciplines and clinicians will certainly be of key importance for greater precision in oncology diagnosis and treatment in the next future.

## Data Availability

All datasets for this study are included in the manuscript and the supplementary files.

## Author Contributions

All authors listed have made a substantial, direct and intellectual contribution to the work, and approved it for publication.

### Conflict of Interest Statement

The authors declare that the research was conducted in the absence of any commercial or financial relationships that could be construed as a potential conflict of interest.
